# Genetic liability for diet-derived circulating antioxidants, oxidative stress, and risk of osteoarthritis: a Mendelian randomization study

**DOI:** 10.3389/fnut.2023.1233086

**Published:** 2023-12-21

**Authors:** Yidan Tang, Xiaolin Xu, Shuangyi Zhang, Weishuang Kong, Weiyi Zhang, Tao Zhu

**Affiliations:** ^1^Department of Anesthesiology, West China Hospital, Sichuan University, Chengdu, China; ^2^Laboratory of Anesthesia and Critical Care Medicine, National-Local Joint Engineering Research Centre of Translational Medicine of Anesthesiology, West China Hospital, Sichuan University, Chengdu, China; ^3^Department of Surgery, Xuanwei Hospital of Traditional Chinese Medicine, Xuanwei, China

**Keywords:** antioxidant, oxidative stress, osteoarthritis, causal effect, Mendelian

## Abstract

**Background:**

Although well-documented, the causal relationships between diet-derived circulating antioxidants, oxidative stress, and osteoarthritis (OA) are equivocal. The objective of this study is to employ two-sample Mendelian randomization (MR) to investigate possible causal relationships among dietary-derived circulating antioxidants, oxidative stress damage indicators, and OA risk.

**Methods:**

Single-nucleotide polymorphisms for diet-derived circulating antioxidants (ascorbate, β-carotene, lycopene, retinol, and α-and γ-tocopherol), assessed as absolute levels and metabolites, as well as oxidative stress injury biomarkers (GSH, GPX, CAT, SOD, albumin, and total bilirubin), were retrieved from the published data and were used as genetic instrumental variables. Summary statistics for gene–OA associations were obtained from publicly available and two relatively large-scale GWAS meta-analyses to date. The inverse-variance weighting method was utilized as the primary MR analysis. Moreover, multivariable MR was used to determine if mediators (BMI and smoking) causally mediated any connection. Furthermore, for each exposure, MR analyses were conducted per outcome database and then meta-analyzed.

**Results:**

Genetically predicted absolute retinol level was causally associated with hip OA risk [odds ratios (ORs) = 0.40, 95% confidence interval (CI) = 0.24–0.68, FDR-corrected *p* = 0.042]. Moreover, genetically predicted albumin level was causally associated with total OA risk (OR = 0.80, 95% CI = 0.75–0.86, FDR-corrected *p* = 2.20E-11), as well as the risk of hip OA (OR = 0.75, 95% CI = 0.68–0.84, FDR-corrected *p* = 1.38E-06) and knee OA (OR = 0.82, 95% CI = 0.76–0.89, FDR-corrected *p* = 4.49E-06). In addition, MVMR confirmed that the effect of albumin on hip OA is independent of smoking initiation, alcoholic drinks per week, and moderate-to-vigorous physical activity levels but may be influenced by BMI.

**Conclusion:**

Evidence from our study supports a potentially protective effect of high levels of retinol and albumin on OA risk.

## 1 Introduction

Osteoarthritis (OA) is a degenerative disease of the entire joint, most notably the knee, but also the hand and hip (1). OA affects ~500 million people globally, and its incidence is steadily increasing due to an aging population and an obesity pandemic, resulting in a substantial public health and health economic burden (1). However, current disease-modifying treatments for OA are minimally effective (2). Therefore, it is necessary to find effective strategies to prevent OA.

OA pathogenesis is regulated by multiple predisposing factors, including imbalanced matrix metabolism, aberrant inflammatory response, and excessive oxidative stress (3). Overproduction of ROS and activation of oxidative stress in chondrocytes are major contributors to OA pathogenesis (4, 5). Furthermore, antioxidants, which can help eliminate free radicals and minimize and eliminate oxidative damage, have been identified as possible targets for the primary prevention of OA (6, 7). The current state of research on the association between antioxidants and OA yields conflicting results. Several studies and reviews analyze the potential protective effects of nutrients, including vitamins D, E, and C, on cartilage metabolism and the development of OA (8, 9). However, some studies have not reported the protective effects of the preceding antioxidants on OA (10, 11). Observational studies are, however, limited by confounding factors and reverse causation bias that led to inconsistent results.

Furthermore, age, gender, obesity, joint biomechanics, history of joint surgery, and genetic susceptibility were identified as the risk factors (12). In addition, recent studies implicated the importance of antioxidants in OA (6, 13). Furthermore, redox equilibrium is maintained by a sophisticated antioxidant defense system comprised of both enzymatic and non-enzymatic antioxidants. The major antioxidant systems in the cell are enzymatic antioxidants, which include glutathione S-transferase (GST), catalase (CAT), superoxide dismutase (SOD), and glutathione peroxidase (GPX). Glutathione (GSH), albumin, and total bilirubin are common non-enzymatic antioxidants. Several studies have reported that the levels of GSH, GPX, CAT, and SOD, as well as total bilirubin, were associated with the OA risk (14–17). The current state of research on the association between albumin and OA yields conflicting results; as a clinical study reported, the concentration of albumin in osteoarthritic knee-joint effusions was distinctly lower than in sera of healthy adults (18), but higher serum albumin levels in OA patients were described in another study (19). Existing observational studies are unable to rule out the effect of reverse causality or unmeasured confounding factors such as socio-economic status, dietary habits, or other health-related behaviors (11, 20, 21). In addition, intervention trials may have limitations due to the potential for unknown risks and harm to subjects. In addition, intervention trials may be confounded by many uncertainty factors such as time, dose, duration, and onset and progression. Therefore, the causal associations between antioxidant levels and the risk of OA remain unknown.

Mendelian randomization (MR) is a viable approach for determining causation by using genetic variations as instrumental variables (IVs) to investigate the causal influence of exposure on outcome. This approach is based on the notion that genetic variations are assigned at random during conception. It is less susceptible to environmental or lifestyle influences, and it decreases the bias caused by reverse causality or confounding factors. This study used antioxidants (including diet-derived circulating antioxidants and their metabolites, as well as oxidative stress injury biomarkers) as exposures and OA as an outcome for MR analysis to investigate the potential causal link to provide a theoretical foundation for future research into the complex processes and risk factors of OA. Moreover, Multivariate Mendelian randomization (MVMR) is a recently established method for assessing independent but related exposures at the same time by including genetic variation for each risk factor in the same model (22). Since MVMR has recently been used to disentangle the direct effects of each risk factor on a variety of health outcomes that are not mediated by other associated risk factors, we used it in this study to assess the potential mediating effects of body mass index (BMI), smoking initiation, alcoholic drinks per week, and moderate-to-vigorous physical activity levels.

## 2 Materials and methods

### 2.1 Study design

**Figure 1:**
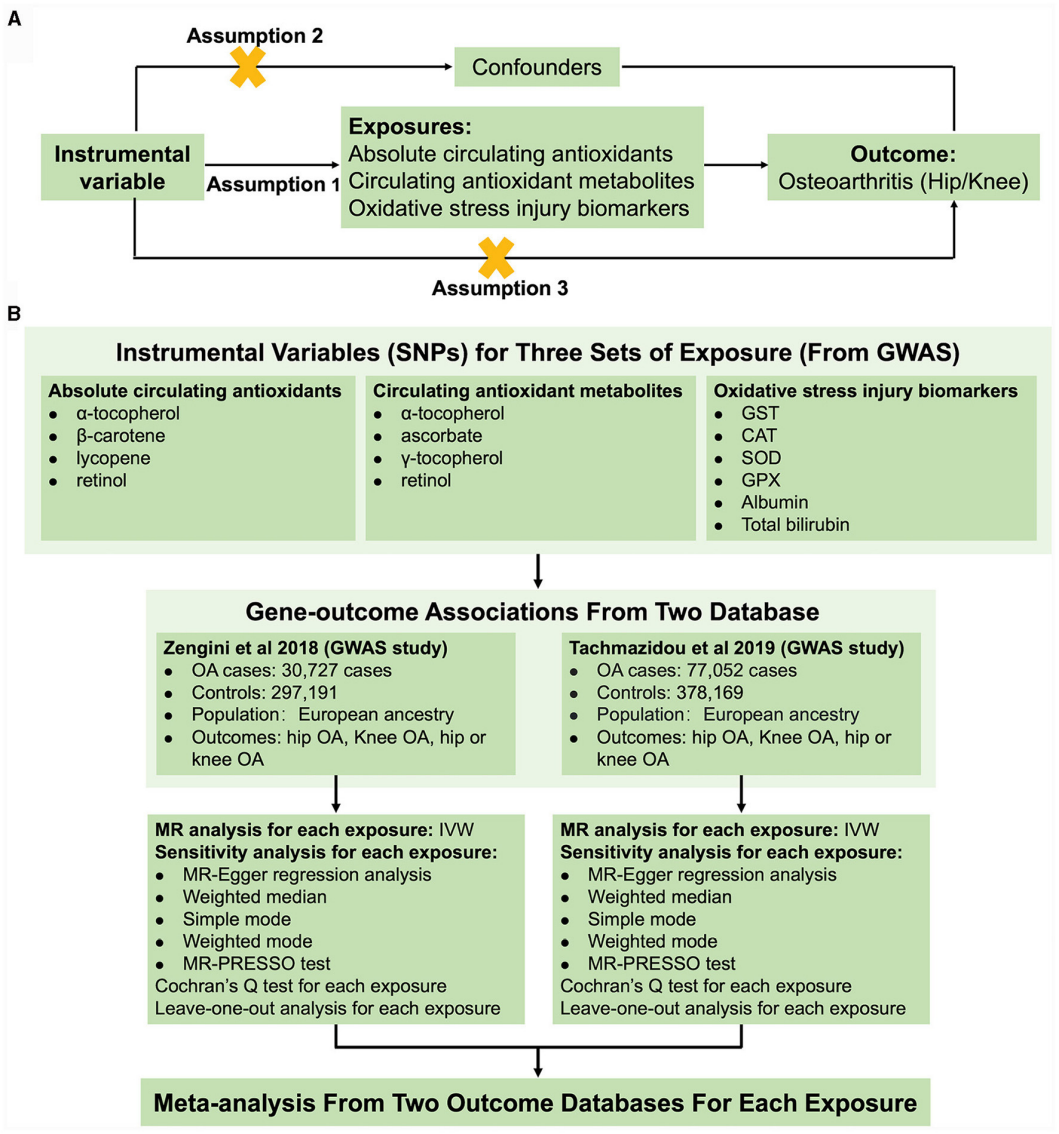
Schematic overview of the study design. **(A)** The MR hypothesis diagram and **(B)** Flowchart of MR analysis and meta-analysis.

For the present study, we performed a two-sample MR to test the associations of absolute circulating levels and corresponding metabolites of antioxidants, as well as oxidative stress injury biomarkers and OA risk. The general design of the current study is illustrated in Figure 1. MR is based on three fundamental assumptions, as genetic instrumental variables should (1) be associated with exposure with the genome-wide significance; (2) not be related to any measured and unmeasured confounders; and (3) not affect the risk of OA through other pathways. All statistics utilized in the present study were derived from publicly available genome-wide association studies (GWAS), and each original study had ethical approval and informed permission. Moreover, our study was conducted based on the MR-STROBE guidance (23).

### 2.2 Selection of genetic instrumental variables

In total, four main diet-derived antioxidants were identified in the present study: vitamin C (ascorbate), β-carotene, lycopene, retinol, and vitamin E (α- and γ-tocopherol). In the present study, both antioxidants that were measured as real absolute blood levels and their related circulating metabolites that were evaluated as relative plasma or serum concentrations were taken into consideration. For absolute antioxidant levels, α-tocopherol, β-carotene, lycopene, and retinol were utilized, whereas for antioxidant metabolites, α-tocopherol, ascorbate, γ-tocopherol, and retinol were included. To identify suitable genetic IVs, a variety of quality control procedures were performed. First, independent single-nucleotide polymorphisms (SNPs) linked with each exposure at genome-wide significance were selected as potential IVs. Second, the linkage disequilibrium (LD) between all SNPs for the same exposure was determined. Finally, to evaluate the strength of the selected SNPs, the F statistics was calculated using the formula: *F* = *R*^2^ (N–k−1)/[(1–*R*^2^) k], where *R*^2^ is the proportion of variability explained by each SNP, N is the sample size of the GWAS, and k is the number of SNPs. When the F-statistic is more than 10, it implies that IV is a strong instrument (24).

### 2.3 Data sources and SNP selection for absolute circulating antioxidants

For α-tocopherol, three SNPs were identified in a GWAS with 4,401 participants (*p* < 5 × 10^−8^, LD < 0.001) (25). Three genetic variants for circulating β-carotene levels were obtained from a GWAS study involving 2,344 subjects in the Nurses' Health Study (*p* < 5 × 10^−8^, LD < 0.2) (26). Five SNPs (*p* < 5 × 10^−6^, LD < 0.001) for circulating lycopene level were identified from a published GWAS study involving 441 older Amish adults (27). Two genetic variants associated with circulating retinol were obtained from a GWAS study of 5,006 Caucasian individuals from two cohorts (*p* < 5 × 10^−8^, LD < 0.001) (28).

### 2.4 Data sources and SNP selection for circulating antioxidant metabolites

Genetic variants of circulating antioxidant metabolites were extracted from recent large-scale GWAS (*p* < 1 × 10^−5^, LD: *r*^2^ = 0.001 and clump distance = 10,000 kb). Eleven instrumental variables of α-tocopherol (*n* = 7,276), 14 instrumental variables of ascorbate (*n* = 2,063), and 13 instrumental variables of γ-tocopherol (*n* = 5,822) were obtained from 7,824 adult individuals from two European population studies (29, 30). A total of 26 SNPs associated with retinol were derived from 1,960 subjects of European descent.

### 2.5 Data sources and SNP selection for oxidative stress injury biomarkers

Genetic predictors for oxidative stress injury biomarkers, such as GST, CAT, GPX, SOD, albumin, and total bilirubin, were derived from the most up-to-date GWAS (*p* < 1 × 10^−5^, LD: *r*^2^ = 0.001 and clump distance = 10,000 kb). Genetic IVs for GST (*n* = 3,301), CAT (*n* = 3,301), SOD (*n* = 3,301), and GPX (*n* = 3,301) were identified in the INTERVAL study (31). Genetic variants of albumin (*n* = 115,06) and total bilirubin (*n* = 342,829) were derived from the UK biobank.

### 2.6 Data sources and SNP selection for OA

As both knee and hip are common sites of OA, summary statistics data on hip, knee, and total OA (hip or knee) were obtained from the publicly available GWAS (32, 33). Zengini et al. performed a GWAS for OA using data across 16.5 million variants from the UK Biobank resource and included 30,727 cases and 297,191 controls. The study by Zengini et al. included self-reported OA and hospital-diagnosed OA patients; the self-reported OA definition includes participants who answered “Yes” to the following question on a touchscreen self-administered questionnaire: “Has a doctor ever told you that you have had any other serious medical conditions or disabilities?;” information on disease code was collected in a subsequent computer-assisted personal interview, and the hospital-diagnosed OA coding in the UK Biobank is based on the ICD-10 code (34). The study by Tachmazidou et al. meta-analyzed the UK Biobank and Arthritis Research UK Osteoarthritis Genetics (arcOGEN) datasets and included 77,052 cases and 378,169 controls. The study by Tachmazidou et al. included the self-reported OA established during an interview with a nurse and the hospital episode statistics ICD10 code for OA in the UK Biobank (34). For the arcOGEN dataset, OA was ascertained based on clinical evidence of disease to a level requiring joint replacement or radiographic evidence of disease (Kellgren–Lawrence grade ≥ 2) (35, 36).

### 2.7 Multivariate MR analysis

As reported in the study, BMI, alcohol consumption, smoking, and physical activity levels may be risk factors for OA and may influence the effect of dietary sources of oxidants on OA (5, 37). To address this issue, we performed MVMR as a sensitivity analysis to correct for measured confounders, such as BMI, smoking initiation, alcoholic drinks per week, and moderate-to-vigorous physical activity levels, which were employed as the potential confounders. For the confounders, we selected the largest published GWAS to date (Table 1).

**Table 1:** Details for selected GWAS of mediators.

**Mediators**	**Sample sizes**	**PMID**
BMI	681,275	30124842
Smoking initiation	607,291	30643251
Alcoholic drinks per week	335,394	30643251
Moderate to vigorous physical activity levels	377,234	29899525

### 2.8 Statistical analysis

In the univariable MR, to explore the causal associations of genetic variants related to exposures on outcomes, the inverse-variance weighting (IVW) method was utilized as the primary analysis for MR. Moreover, MR-Egger, simple mode, weighted median, and weighted mode models were employed as sensitivity analysis methods to test the robustness of the results. If trustworthy instruments are provided by SNPs accounting for at least 50% of the weight, then the weighted median estimate generates valid results as it is the median of the SNP-specific estimates. Even if all genetic variants are invalid, the MR-Egger regression can estimate the underlying causal impact. In addition, the weighted mode approach remains viable even if the other instrumental variables do not meet the MR method's prerequisites for causal inference as long as the majority of IVs have equal causal estimations. Horizontal pleiotropy is defined as some instruments influencing the outcome via paths that bypass the exposure (38). The directionality of pleiotropy can be detected and adjusted using the MR-Egger technique based on the Instrument Strength Independent of Direct Effect (InSIDE) assumption, although it is underpowered (39). Additionally, the MR Pleiotropy RESidual Sum and Outlier (MR-PRESSO) test was used to detect potential horizontal pleiotropy and reduce the effects of pleiotropy by eliminating outliers (40). The heterogeneity was assessed using Cochran's Q-statistic. The leave-one-out analysis was used to see if any of the significant results were impacted by a specific SNP. Results are expressed as con OA risk for a corresponding unit change in absolute circulating antioxidants levels of α-tocopherol (mg/L in log-transformed scale), lycopene (μg/dL), β-carotene (μg/L in natural log-transformed scale), and retinol (μg/L in natural log-transformed scale), or a 10-fold change in circulating antioxidant metabolites concentrations. For analyses with biomarkers level as the exposure, we provide the odds ratios (OR) for OA associated with each standard deviation (SD) increase in levels of biomarkers for oxidative stress damage. For MVMR analysis, the inverse-variance weighted method was employed.

All exposure-specific MR analyses were performed independently in the studies by Tachmazidou et al. (33) and Zengini et al. (32) and then meta-analyzed to obtain pooled estimates for each exposure on OA risk. We used *I*^2^ statistics to measure heterogeneity between estimates from two studies and the associated *p*-value from Cochran's Q test. When there is no heterogeneity, the fixed-effect model meta-analyses are used to pool instrumental variable estimates across the two outcome databases for each exposure, while random-effect model meta-analyses are employed when there is heterogeneity.

A *p*-value of < 0.05 was regarded as suggestive evidence for a possible causal relationship. To account for multiple testing (many exposures), the statistical significance of the MR effect estimates was set at < 5% using the Benjamini–Hochberg false discovery rate (FDR). All analyses were carried out in R version 4.3.0 using the packages “TwoSampleMR,” “MRPRESSO,” and “meta.”

## 3 Results

### 3.1 Screening of genetic instrumental variables

The features of genetic instruments identified for dietary-derived antioxidants as absolute levels and metabolites, as well as oxidative stress damage biomarkers, are summarized in Table 2. Supplementary Tables 1–3 provide detailed information on the variants, their associations with antioxidants (beta_gene − exposure_), oxidative stress injury biomarkers, and with OA (beta_gene − outcome_) across databases. The F-statistics for all genetic instruments employed in this study were >10, demonstrating that the IVs were strong instruments, lowering the bias of IV estimates.

**Table 2:** Details of GWAS studies for diet-derived circulating antioxidants and oxidative stress-related traits.

**Trait**	**Numbers of SNPs**	***p*-value**	**Unit**	***R^2^***	**F statistic**	**Participants (Numbers)**	**PMID**
**Absolute circulating antioxidants**							
α-tocopherol	3	5 × 10^−8^	mg/L in log-transformed scale	0.017	86.54	4,014	21729881
Lycopene	5	5 × 10^−6^	μg/dL	0.301	189.04	441	26861389
β-carotene	3	5 × 10^−8^	μg/L in natural log-transformed scale	0.09	231.63	2,344	23134893
Retinol	2	5 × 10^−8^	μg/L in natural log-transformed scale	0.023	117.8	5,006	21878437
**Circulating antioxidants metabolites**							
α-tocopherol	11	1 × 10^−5^	log10-transfomed metabolites concentration	0.033	198.61	7,276	24816252
γ-tocopherol	13	1 × 10^−5^	log10-transfomed metabolites concentration	0.15	1027.06	5,822	24816252
Retinol	24	1 × 10^−5^	log10-transfomed metabolites concentration	0.048	98.57	1,957	28263315
Ascorbate	14	1 × 10^−5^	log10-transfomed metabolites concentration	0.186	470.94	2,063	24816252
**Oxidative stress injury biomarkers levels**							
GST	14	1 × 10^−5^	-	0.013	45.89	3,301	29875488
CAT	27	1 × 10^−5^	-	0.007	24.89	3,301	29875488
SOD	23	1 × 10^−5^	-	0.008	25.14	3,301	29875488
GPX	22	1 × 10^−5^	-	0.011	38.15	3,301	29875488
Albumin	107	1 × 10^−5^	-	0.0004	42.20	115,060	-
Total bilirubin	360	1 × 10^−5^	-	0.001	418.71	342,829	-

### 3.2 Absolute circulating antioxidants and OA

**Figure 2:**
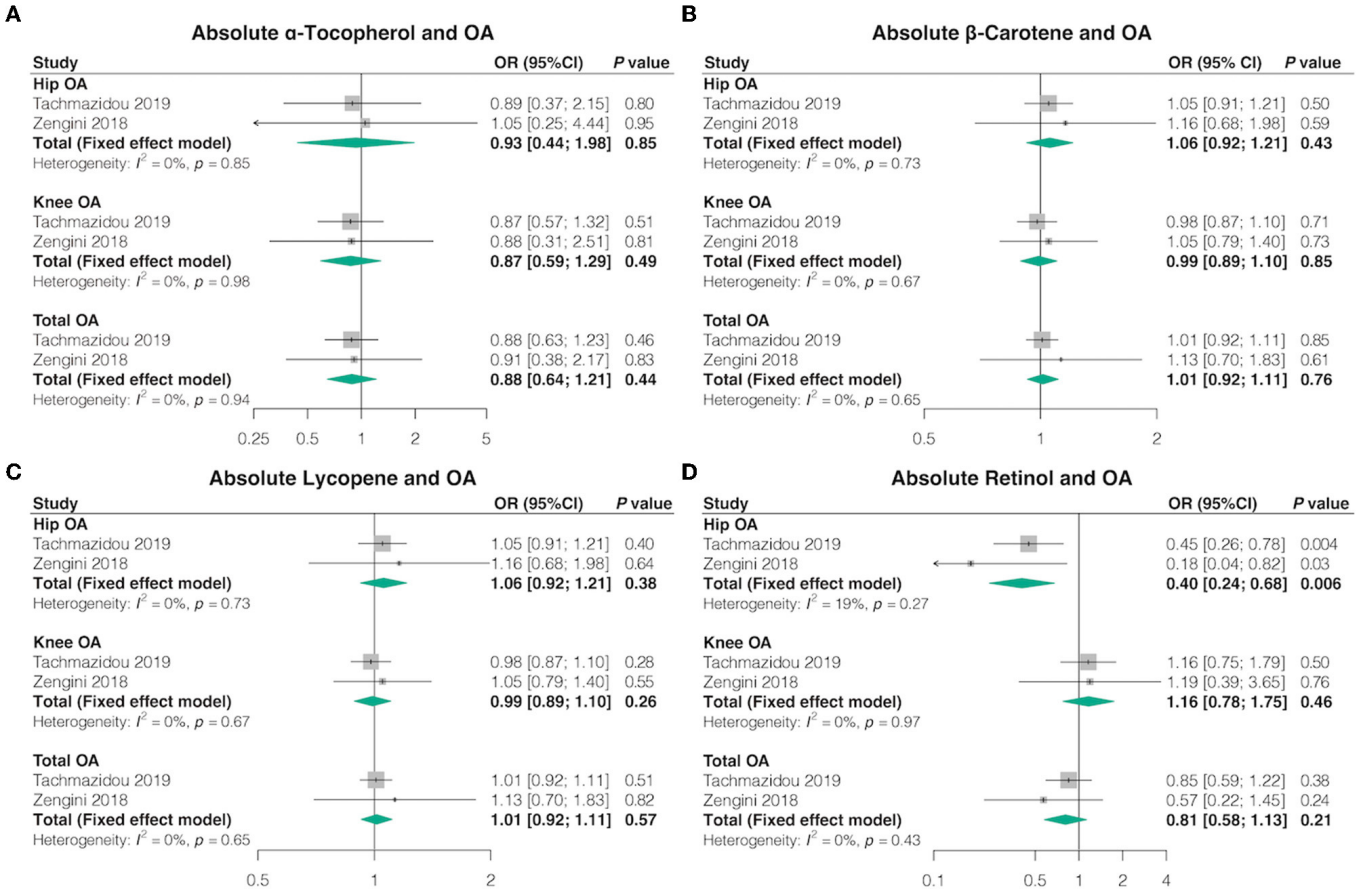
Causal association between absolute circulating antioxidants with OA. The effect of **(A)** absolute α-tocopherol, **(B)** absolute β-carotene, **(C)** absolute lycopene, and **(D)** absolute retinol on OA risk.

Overall, in the univariable MR analyses using IVW, most genetically determined absolute dietary-derived antioxidant levels were not associated with the risk of OA in both databases, but genetically predicted absolute retinol levels were associated with lower odds hip OA [OR = 0.40, 95% confidence interval (CI) = 0.24–0.68, *p* = 0.006, FDR-corrected *p* = 0.042] (Figure 2; Supplementary Tables 5, 6). Pooled odds ratio (OR) for hip OA per unit increase of antioxidants was 0.93 (95% CI = 0.44–1.98, *p* = 0.85, FDR-corrected *p* = 0.85) for log-transformed α-tocopherol, 1.06 (95% CI = 0.92–1.21, *p* = 0.43, FDR-corrected *p* = 0.62) for natural log-transformed β-carotene, and 1.06 (95% CI = 0.92–1.21, *p* = 0.38, FDR-corrected *p* = 0.62) for 1 μg/dl lycopene. Pooled OR for knee OA per unit increase of antioxidants was 0.87 (95% CI = 0.59–1.29, *p* = 0.49, FDR-corrected *p* = 0.69) for log-transformed α-tocopherol, 0.99 (95% CI = 0.89–1.10, *p* = 0.85, FDR-corrected *p* = 0.86) for natural log-transformed β-carotene, 0.99 (95% CI = 0.89-1.10, *p* = 0.26, FDR-corrected *p* = 0.46) for 1 μg/dl lycopene, and 1.16 (95% CI = 0.78–1.75, *p* = 0.46, FDR-corrected *p* = 0.69) for natural log-transformed retinol. Pooled OR for total OA per unit increase of anti-oxidants was 0.88 (95% CI = 0.64–1.21, *p* = 0.44, FDR-corrected *p* = 0.61) for log-transformed α-tocopherol, 1.01 (95% CI = 0.92–1.11, *p* = 0.76, FDR-corrected *p* = 0.76) for natural log-transformed β-carotene, 1.01 (95% CI = 0.92–1.11, *p* = 0.57, FDR-corrected *p* = 0.61) for 1 μg/dl lycopene, and 0.81 (95% CI = 0.58–1.13, *p* = 0.21, FDR-corrected *p* = 0.49) for natural log-transformed retinol.

We obtained similar outcomes using additional MR estimation methods (MR-Egger, weighted median, simple mode, and weighted mode) (Supplementary Tables 4, 5). Basically, no heterogeneity was detected by Cochran's Q test, but there was considerable heterogeneity between lycopene and hip OA from the study by Zengini et al. (Q = 47.25; *p* = 0.04) (Supplementary Table 6). MR-Egger regression analysis suggested no evidence of directional pleiotropy for the IVs (Supplementary Table 7). The MR-PRESSO test uncovered no evidence of horizontal pleiotropy in the relationships between absolute dietary-derived antioxidant levels and OA (Supplementary Table 8). In addition, the leave-one-out analysis indicated that the most causal association signals were not driven by any single SNP (Supplementary Table 9).

### 3.3 Circulating antioxidant metabolites and OA

**Figure 3:**
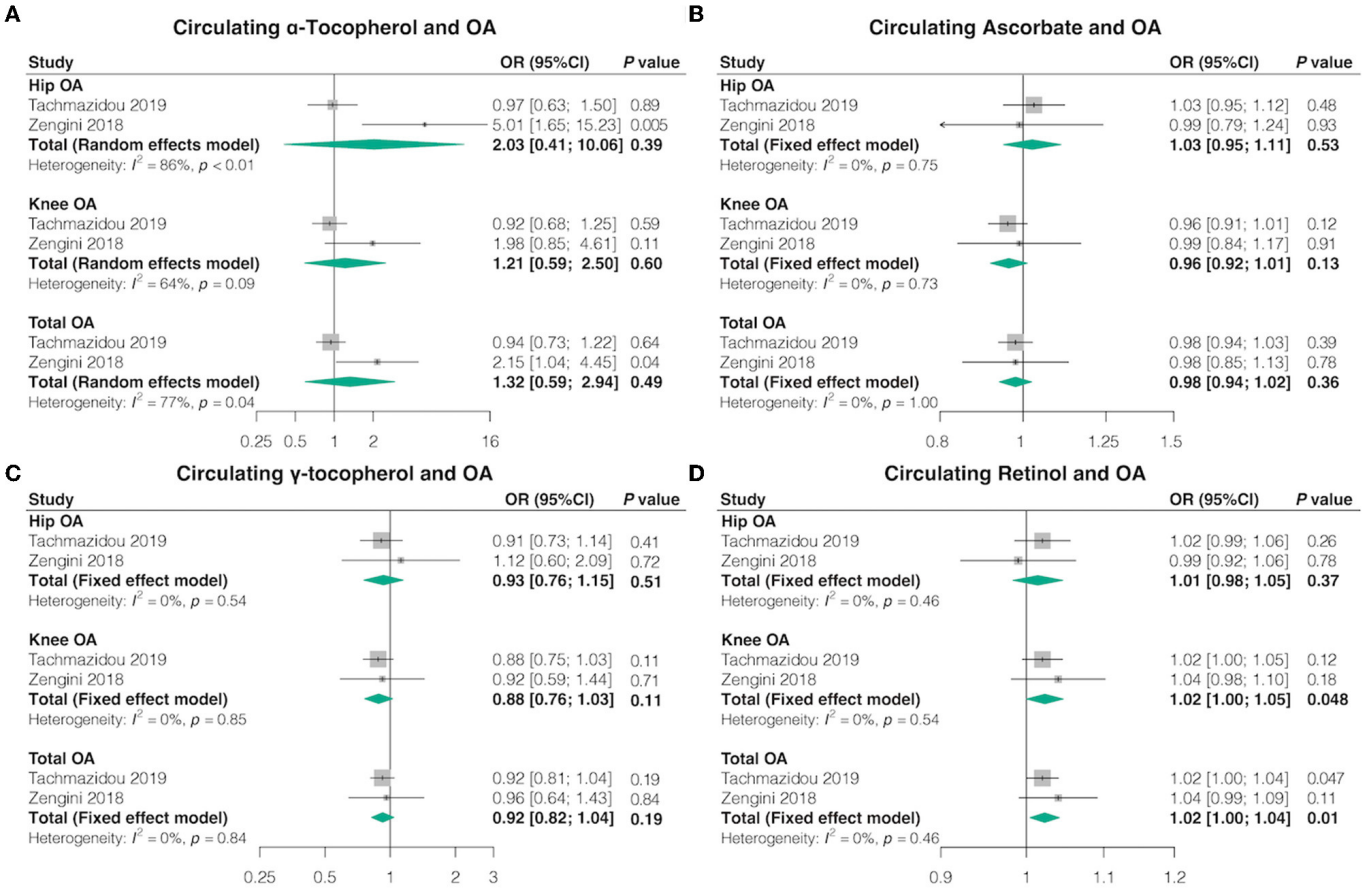
Causal association between circulating antioxidant metabolites with OA. The effect of **(A)** circulating α-tocopherol, **(B)** circulating ascorbate, **(C)** circulating γ-tocopherol, and **(D)** circulating retinol on OA risk.

In the univariable MR, consistent with the findings from absolute circulating anti-oxidants, most genetically determined absolute dietary-derived antioxidant levels were not associated with the risk of OA in both databases, but genetically predicted circulating retinol levels were associated with higher odds knee OA (OR = 1.02, 95% CI = 1.00–1.05, *p* = 0.048, FDR-corrected *p* = 0.30) and total OA (OR = 1.02, 95% CI = 1.00–1.04, *p* = 0.01, FDR-corrected *p* = 0.07) (Figure 3; Supplementary Tables 10, 11). The combined ORs for hip OA per 10-fold increase in metabolites concentration were 2.03 (95% CI = 0.41–10.06, *p* = 0.39, FDR-corrected *p* = 0.62) for α-tocopherol, 1.03 (95% CI = 0.95–1.11, *p* = 0.53, FDR-corrected *p* = 0.62) for ascorbate, 0.93 (95% CI = 0.76–1.15, *p* = 0.51, FDR-corrected *p* = 0.62) for γ-tocopherol, and 1.01 (95% CI = 0.98–1.05, *p* = 0.37, FDR-corrected *p* = 0.62) for retinol. The combined ORs for knee OA per 10-fold increase in metabolites concentration were 1.21 (95% CI = 0.59–2.50, *p* = 0.60, FDR-corrected *p* = 0.76) for α-tocopherol, 0.96 (95% CI = 0.92–1.01, *p* = 0.13, FDR-corrected *p* = 0.30) for ascorbate, and 0.88 (95% CI = 0.76–1.03, *p* = 0.11, FDR-corrected *p* = 0.30) for γ-tocopherol. The combined ORs for total OA per 10-fold increase in metabolites concentration were 1.32 (95% CI = 0.59–2.94, *p* = 0.49, FDR-corrected *p* = 0.61) for α-tocopherol, 0.98 (95% CI = 0.94–1.02, *p* = 0.36, FDR-corrected *p* = 0.61) for ascorbate, and 0.92 (95% CI = 0.82–1.04, *p* = 0.19, FDR-corrected *p* = 0.61) for γ-tocopherol.

Furthermore, we achieved similar outcomes utilizing additional MR estimation methods (MR-Egger, weighted median, simple mode, and weighted mode) (Supplementary Tables 10, 11). Cochran's Q test revealed no heterogeneity for circulating antioxidant metabolites IVs in three OA types (hip, knee, and total OA) but considerable heterogeneity between γ-tocopherol and total OA from the study by Zengini et al. (Q = 47.25; *p* = 0.04) (Supplementary Table 12). MR-Egger regression analysis suggested no evidence of directional pleiotropy for the IVs (Supplementary Table 13). MR-PRESSO test uncovered no evidence of horizontal pleiotropy in the relationship between absolute dietary-derived antioxidant levels and OA (Supplementary Table 9). In addition, the leave-one-out analysis indicated that there were SNPs with potential effects on the pooled results, suggesting the need for careful interpretation of the causal association signals (Supplementary Table 14).

### 3.4 Oxidative stress injury biomarkers and OA

**Figure 4:**
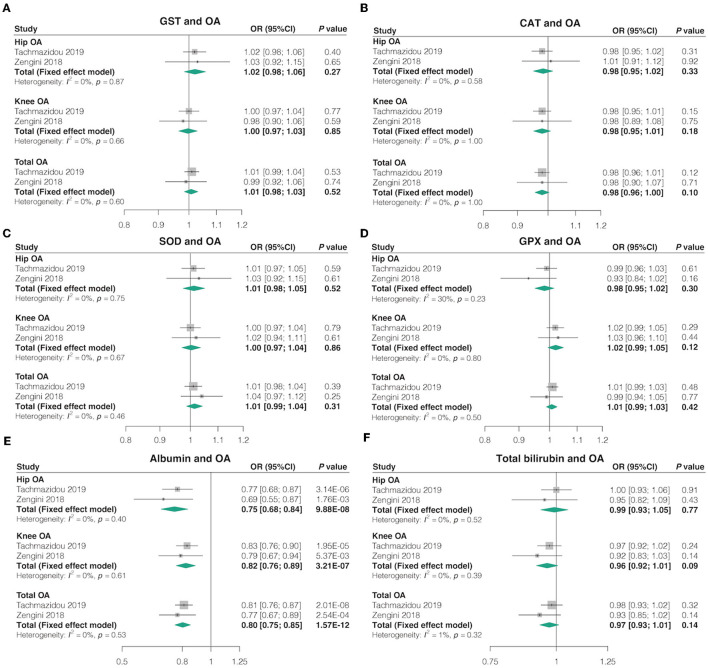
Causal associations between oxidative stress injury biomarkers and OA. The effect of **(A)** GST, **(B)** CAT, **(C)** SOD, **(D)** GPX, **(E)** albumin, and **(F)** total bilirubin absolute retinol on OA risk.

Overall, in the univariable MR using IVW, most genetically determined oxidative stress injury biomarkers levels were not associated with the risk of OA in both databases, except each SD higher albumin level was associated with lower odds hip OA (OR = 0.75, 95% CI = 0.68–0.84, *p* = 9.88 × 10-8, FDR-corrected *p* = 1.38 × 10^−6^), lower odds knee OA (OR = 0.82, 95% CI = 0.76–0.89, *p* = 3.21 × 10-7, FDR-corrected *p* = 4.49 × 10^−6^), and lower odds total OA (OR = 0.80, 95% CI = 0.75–0.86, p = 1.575 × 10-12, FDR-corrected *p* = 2.20 × 10-11) (Figure 4; Supplementary Tables 15, 16). The combined ORs for hip OA in each SD higher oxidative stress injury biomarkers levels were 1.02 (95% CI = 0.98–1.06, *p* = 0.27, FDR-corrected *p* = 0.62) for GST, 0.98 (95% CI = 0.95–1.02, *p* = 0.33, FDR-corrected *p* = 0.62) for CAT, 1.01 (95% CI = 0.98–1.05, *p* = 0.52, FDR-corrected *p* = 0.62) for SOD, 0.98 (95% CI = 0.95–1.02, *p* = 0.30, FDR-corrected *p* = 0.62) for GPX, and 0.99 (95% CI = 0.93–1.05, *p* = 0.77, FDR-corrected *p* = 0.83) for total bilirubin. The combined ORs for knee OA in each SD higher oxidative stress injury biomarkers levels were 1.00 (95% CI = 0.97–1.03, *p* = 0.85, FDR-corrected *p* = 0.86) for GST, 0.98 (95% CI = 0.95–1.01, *p* = 0.18, FDR-corrected *p* = 0.36) for CAT, 1.00 (95% CI = 0.97–1.04, *p* = 0.86, FDR-corrected *p* = 0.86) for SOD, 1.02 (95% CI = 0.99–1.05, *p* = 0.12, FDR-corrected *p* = 0.30) for GPX, and 0.96 (95% CI = 0.92–1.01, *p* = 0.09, FDR-corrected *p* = 0.30) for total bilirubin. The combined ORs for total OA in each SD higher oxidative stress injury biomarkers levels were 1.01 (95% CI = 0.98–1.03, *p* = 0.52, FDR-corrected *p* = 0.61) for GST, 0.98 (95% CI = 0.96–1.00, *p* = 0.10, FDR-corrected *p* = 0.47) for CAT, 1.01 (95% CI = 0.98–1.05, *p* = 0.52, FDR-corrected *p* = 0.61) for SOD, 0.98 (95% CI = 0.95–1.02, *p* = 0.30, FDR-corrected *p* = 0.61) for GPX, and 0.97 (95% CI = 0.93–1.01, *p* = 0.14, FDR-corrected *p* = 0.49) for total bilirubin (Figure 4).

In addition, similar findings were observed when using other MR estimate methods, such as MR-Egger, weighted median, simple mode, and weighted mode (Supplementary Table 16). Cochran's Q test revealed no heterogeneity for circulating antioxidant metabolites IVs in three OA types (hip, knee, and total OA) but considerable heterogeneity for the effects of albumin, CAT, and total bilirubin on OA (Supplementary Table 17). Basically, no directional pleiotropy was detected by MR-Egger regression analysis (all *P*-values for intercept > 0.05), but potential directional pleiotropy between total bilirubin and OA (Supplementary Table 18). The MR-PRESSO test detected some outliers for the effects of CAT, albumin, and total bilirubin on OA, and the causal effects remained unchanged after the removal of outliers SNPs (Supplementary Table 19). In addition, the leave-one-out analysis indicated that the most causal association signals were not driven by any single SNP, but the potential effect of the total bilirubin on hip OA (from the study by Tachmazidou et al.) may be influenced by some potential SNPs (Supplementary Table 20).

### 3.5 Exploration of the potential confounding factors

In the MVMR analysis, after adjusting for smoking initiation, alcoholic drinks per week, and moderate-to-vigorous physical activity levels, the IVW results of MVMR analyses demonstrated that each SD higher albumin level was significantly correlated with the lower risk of hip OA (Table 3). However, the effect of albumin on hip OA was substantially reduced in MVMR analyses incorporating BMI (Table 3). In addition, there are not enough SNPs for MVMR analysis of absolute retinol.

**Table 3:** Causal effect of albumin on hip OA in multivariable Mendelian randomization analyses.

**Mediators adjusted**	**Effect of albumin on Hip OA** **(****33****)**		**Effect of albumin on Hip OA** **(****32****)**	
	**Effect estimate (IVW): OR (95% CI)**	***p***	**Effect estimate (IVW): OR (95% CI)**	***p***
BMI	0.93 (0.79, 1.09)	0.09	0.88 (0.60, 1.3)	0.63
Smoking initiation	0.78 (0.70, 0.87)	1.27 × 10^−5^	0.76 (0.58, 0.98)	0.04
Alcoholic drinks per week	0.78 (0.69, 0.87)	1.91 × 10^−5^	0.68 (0.54, 0.86)	0.001
Moderate to vigorous physical activity levels	0.80 (0.72, 0.89)	2.55 × 10^−5^	0.74 (0.58, 0.95)	0.02

## 4 Discussion

In the present study, we investigated the causal associations between dietary-derived antioxidants/oxidative stress injury biomarkers and OA risk using MR analysis with the large publicly available GWAS datasets. Our findings indicate that the levels of absolute retinol circulating retinol and albumin may be causative factors of OA risk. Moreover, MVMR confirmed that the effect of albumin on hip OA is independent of smoking initiation, alcoholic drinks per week, and moderate-to-vigorous physical activity levels but may be influenced by BMI.

There has been obvious controversy over whether antioxidants reduce the risk of OA. Our study found that there was no causal relationship between vitamin E (α- and γ-tocopherol), β-Carotene, and lycopene levels and the risk of OA. Previous clinical trials have suggested that oral vitamin E may reduce pain in OA patients (41, 42). In addition, a cross-sectional investigation in a community-based study in Japan found that high serum values of γ-tocopherols were significantly associated with a low risk of radiographic knee OA (43). However, a double-blind, randomized, placebo-controlled study reported that vitamin E was not effective in relieving symptoms of knee OA (44), and a cross-sectional study reported that the vitamin E intake tended to be positively associated with the tibial plateau bone area, which is a negative effect on the bone and might even increase the OA risk (21). γ-Tocopherol is often the most prevalent form of vitamin E in plant seeds, and α-tocopherol is the predominant form of vitamin E in most human and animal tissues, including blood plasma. In addition, in a prospective cohort study, higher β-Carotene intake was associated with a reduced prevalence of femoral head bone marrow lesions in OA patients (45). In contrast, some cross-sectional studies described that carotenoid, including β-Carotene intake, was not significantly associated with cartilage or bone measures or the risk of OA (21, 43). Interestingly, a case–control study reported that the people with the highest levels of trans-beta-carotene were more likely to have knee OA (46). A cross-sectional study observed that vitamin C intake was inversely associated with the tibial plateau bone area and with the presence of bone marrow lesions, both of which are important in the pathogenesis of knee OA (21). However, a cohort study observed that there was no association between the dietary vitamin C and the risk of OA (11). A prospective cohort study in Australia reported that higher lycopene intake was associated with a reduced prevalence of femoral head cartilage defects in community-based adults (45). In addition, a case–control study indicated that lower serum lycopene levels were associated with higher pain and physical disability in knee OA patients (47). These disparities might be attributed to eating habits, subject characteristics, and sample size (48).

Retinol, a form of vitamin A, is a fat-soluble vitamin in the vitamin A family found in food and used as a dietary supplement. Moreover, our pooled results from two databases indicated that absolute retinol level was negatively correlated with hip OA risk, while circulating retinol was positively correlated with knee OA and total OA (hip or knee). A recent MR study reported the inverse causal association between absolute serum retinol levels and hip OA utilizing one GWAS, which is consistent with our pooled results from two databases in our study (49). In addition, a longitudinal cohort study over a 10-year follow-up period recently described that women with low serum retinol levels develop knee OA at a significantly lower rate among community-dwelling people in Japan (50). Interestingly, a cross-sectional investigation in a community-based study reported no associations between serum retinol level and the development of radiographic knee OA in rural Japanese inhabitants (43). Existing research on the role of vitamin A in OA is highly controversial. Some studies reported that the effect of vitamin A on OA was negative (51). There is even evidence that vitamin A metabolite levels are elevated in synovial fluid, serum, and cartilage from patients with OA, and they appear to drive OA development (52, 53).

Enzymatic antioxidants such as GST, CAT, SOD, and GPX can limit peroxide generation and eliminate free radicals (54). Several studies have reported OA disease models with lower GSH, GPX, CAT, and SOD levels (14–16), as well as higher total bilirubin levels (17). Interestingly, other studies observed no significant difference in the GSH level, as well as the activity of SOD and GPX between OA patients and control people was insignificantly lower than in healthy people (55, 56). Non-enzymatic antioxidants, including albumin and bilirubin, which break the free radical chain reaction interacting with ROS, also play a key role in the antioxidant system (54). Approximately 5% human serum albumin has been reported to reduce pain and effectively treat OA in patients by increasing anti-inflammatory prostaglandins, promoting inflammation and healing as well as cartilage regeneration (57). Our results support the finding and indicate that a high albumin level was associated with a lower risk of OA. Yet, some studies reported no significant differences in plasma and synovial fluid albumin levels between OA patients and healthy people, which may be limited by the small sample sizes (58).

The present study has several strengths. First, MR uses genetic variation as a proxy for environmental exposure to determine the causal relationship between exposure and disease outcome. Genetic differences are assumed to be randomly assigned before birth, are highly independent of environmental variables such as dietary patterns and lifestyle, and are established before the onset of disease. Therefore, MR analyses using genetic variation as an instrumental variable for exposure may reduce the bias of causal reversals and confounders common in observational studies. Second, we further restricted the inclusion of populations of European ancestry to minimize population stratification. In addition, we conducted a meta-analysis of two large databases, and the results from these two databases were largely consistent with weak evidence of heterogeneity, supporting the robustness of our findings. Thus, despite the limited number of robust genetic instruments, the precision of the final MR estimates and the reliability of the results were significantly improved.

However, some limitations concerning the interpretation of the results should also be considered. First, we were unable to test for a non-linear causal link between antioxidant levels and OA risk since the published data we used were summary-level statistics. Second, sensitivity analysis could not be performed due to the limited number of SNPs for the absolute retinol instrumental variable, and a large GWAS of antioxidants will be required in future to increase the instrument's variable strength. Third, the lack of demographic data, such as gender and ethnicity, and other relevant characteristics in the original study prevented additional subgroup analyses. Finally, we were unable to explore the association between antioxidant use and risk of OA in nutritionally deficient populations that may be more interested in supplementing with antioxidants or testing the effects of antioxidants in combination with other treatments. Furthermore, due to the availability of data, this study focused on populations of European origin, and further validation of associations in other populations is needed.

Evidence from the present study supports the beneficial role of absolute circulating retinol levels and albumin in OA. Dietary retinol and albumin supplementation may be a useful strategy for the primary prevention of OA in high-risk individuals.

## Data availability statement

The original contributions presented in the study are included in the article/Supplementary material, further inquiries can be directed to the corresponding authors.

## Author contributions

YT, WZ, and TZ mainly designed the study. YT performed analysis, verified data, and wrote the manuscript. XX, SZ, and WK analyzed data, performed the literature search, and wrote the manuscript. WZ and TZ supervised the manuscript. All authors have read, provided critical feedback on intellectual content, and approved the final manuscript.
