# Corrigendum: Genetic liability for diet-derived circulating antioxidants, oxidative stress, and risk of osteoarthritis: a Mendelian randomization study

**DOI:** 10.3389/fnut.2024.1370331

**Published:** 2024-02-16

**Authors:** Yidan Tang, Xiaolin Xu, Shuangyi Zhang, Weishuang Kong, Weiyi Zhang, Tao Zhu

**Affiliations:** ^1^Department of Anesthesiology, West China Hospital, Sichuan University, Chengdu, China; ^2^Laboratory of Anesthesia and Critical Care Medicine, National-Local Joint Engineering Research Centre of Translational Medicine of Anesthesiology, West China Hospital, Sichuan University, Chengdu, China; ^3^Department of Surgery, Xuanwei Hospital of Traditional Chinese Medicine, Xuanwei, China

**Keywords:** antioxidant, oxidative stress, osteoarthritis, causal effect, Mendelian

In the published article, there was an error in Figure 1 as published. The sample size for the Zengini et al. study was incorrectly written as “77,052 cases and 378,169 controls”. The correct sample size is “30,727 cases and 297,191 controls” has been written incorrectly. The corrected Figure 1 and its caption appear below.

**Figure 1:**
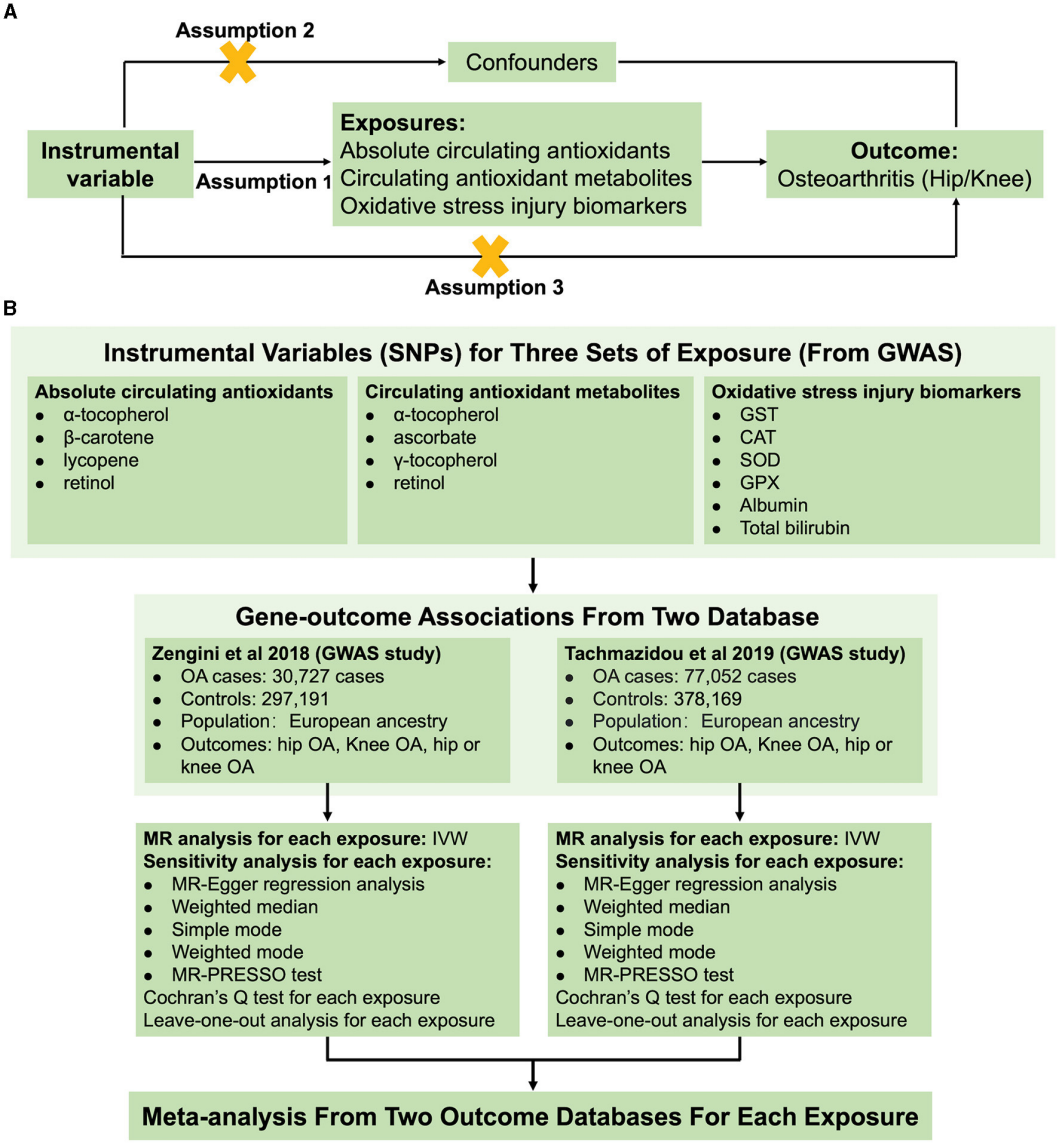
Schematic overview of the study design. **(A)** The MR hypothesis diagram and **(B)** Flowchart of MR analysis and meta-analysis.

The authors apologize for this error and state that this does not change the scientific conclusions of the article in any way. The original article has been updated.

